# Ablation of the Presynaptic Protein Mover Impairs Learning Performance and Decreases Anxiety Behavior in Mice

**DOI:** 10.3390/ijms231911159

**Published:** 2022-09-22

**Authors:** Eva Maria Schleicher, Thomas A. Bayer, Trendelina Iseni, Frederik Wilhelm Ott, Jannek Moritz Wagner, Julio S. Viotti, Thomas Dresbach, Yvonne Bouter

**Affiliations:** 1 Department of Psychiatry and Psychotherapy, Division of Molecular Psychiatry, University Medical Center, Georg-August University, 37075 Goettingen, Germany; 2 Department of Internal Medicine I, University Medical Center of the Johannes Gutenberg University, 55131 Mainz, Germany; 3 Cirrhosis Center Mainz (CCM), University Medical Center of the Johannes Gutenberg University, 55131 Mainz, Germany; 4 Institute of Anatomy and Embryology, University Medical Center Goettingen, Georg-August University, 37075 Goettingen, Germany; 5 Department of Nuclear Medicine, University Medical Center Göttingen, 37075 Goettingen, Germany

**Keywords:** mover, knockout mice, behavior, spatial learning, hippocampus, synaptic facilitation, exploratory behavior, SNARE, synaptic vesicles

## Abstract

The presynaptic protein Mover/TPRGL/SVAP30 is absent in *Drosophila* and *C. elegans* and differentially expressed in synapses in the rodent brain, suggesting that it confers specific functions to subtypes of presynaptic terminals. In order to investigate how the absence of this protein affects behavior and learning, Mover knockout mice (KO) were subjected to a series of established learning tests. To determine possible behavioral and cognitive alterations, male and female 8-week-old KO and C57Bl/6J wildtype (WT) control mice were tested in a battery of memory and anxiety tests. Testing included the cross maze, novel object recognition test (NOR), the Morris water maze (MWM), the elevated plus maze (EPM), and the open field test (OF). Mover KO mice showed impaired recognition memory in the NOR test, and decreased anxiety behavior in the OF and the EPM. Mover KO did not lead to changes in working memory in the cross maze or spatial reference memory in the MWM. However, a detailed analysis of the swimming strategies demonstrated allocentric-specific memory deficits in male KO mice. Our data indicate that Mover appears to control synaptic properties associated with specific forms of memory formation and behavior, suggesting that it has a modulatory role in synaptic transmission.

## 1. Introduction

The formation and maturation of neuronal circuits during development, and the subsequent processes of memory formation, depend crucially on the dynamic regulation of excitability and synaptic transmission [1]. Synaptic transmission is initiated by the exocytosis of neurotransmitters. Neurotransmitter release relies on sets of evolutionary conversed proteins that mediate exocytosis, retrieval, and the re-use of neurotransmitter containing synaptic vesicles (SVs) [2]. Most of the molecules mediating SV exocytosis at active zones are functionally and structurally highly conserved.

Mover is a small protein (266 amino acids) of the presynaptic machinery, which is found on SVs [3]. It was first identified as a binding partner of the scaffolding protein Bassoon in a yeast-2-hybrid assay [3,4]. Because of its unclear function, the protein was initially named in the context of its environment: synaptic vesicle-associated protein of 30kDa (SVAP30), transformation-related protein 63 regulated like (TPRGL, TPRG1L), and family with sequence similarity 79 (FAM79a) [5].

Mover is controlled by the transcription factor P73, which is involved in brain development. Therefore, Mover might play a role in early development independently of its function during synaptic transmission [6]. Quantitative analysis revealed that the amount of Mover relative to the amounts of the general SV marker protein synaptophysin varies among synapses in the brain [7]. One extreme example of this is the fact that inhibitory synapses in the hippocampal CA3 region lack Mover, while excitatory synapses in the same region contain Mover [3]. In addition to its expression in the central nervous system, Mover is also found in several other tissues, such as the skin, liver, and testis [3,6]. To address how the absence of Mover affects behavior and learning in mice lacking Mover, knockout mice were subjected to a series of established learning tests.

## 2. Results

### 2.1. Impaired Recognition Memory in Mover Knockout Mice

The cognitive behavior of Mover KO and WT mice was tested in the cross maze, novel object recognition task (NOR), and Morris water maze (MWM), which measure working memory, recognition memory, and spatial reference memory, respectively.

**Figure 1:**
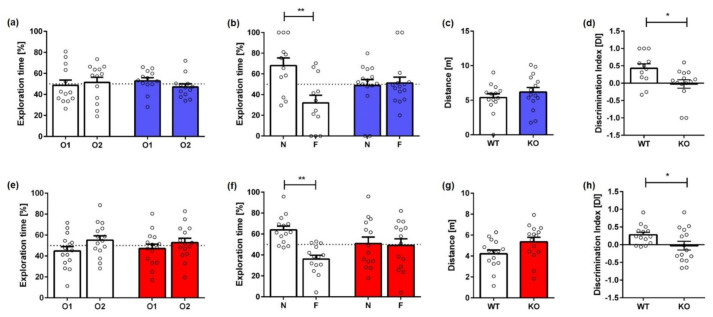
**Recognition memory deficits of Mover KO mice in the novel object recognition task.** During the training phase, all male (**a**) and female (**e**) mice spent equal amounts of time with two similar objects (O1, O2). During the testing phase, only male (**b**) and female WT animals (**f**) showed a significant preference for the novel object (N). In contrast, Mover KO mice did not discriminate between the novel (N) and the familiar object (F). Distance traveled did not differ between male (**c**) or female KO (**g**) and WT animals. Calculation of the discrimination index (DI) also revealed recognition memory deficits in male (**d**) and female KO mice (**h**). Fifty percent chance level is indicated by a dashed line. Paired *t*-test (**a**,**b**,**e**,**f**) and unpaired *t*-test (**c**,**d**,**g**,**h**); *n* = 15–17. Data presented as mean ± S.E.M. * *p* < 0.05; ** *p* < 0.01.

Recognition memory was assessed using the NOR (Figure 1). KO mice showed impaired recognition memory, as they were unable to distinguish between a new (N) and familiar (F) object. During the exploration phase on the first day, all mice spent equal amounts of time exploring each of the similar objects (Figure 1a,e; two-way repeated measures ANOVA, *male*: F(1,25) = 0.5498, *p* = 0.4656; *female*: F(1,28) = 1.852, *p* = 0.1845). When tested for recognition memory 24 h later, male WT mice showed a significant preference towards the novel object (Figure 1b,f; two-way repeated measures ANOVA, *male*: F(1,25) = 4.381, *p* = 0.0466; Bonferroni comparisons: **WT** *N vs. F*: *p* = 0.0287). In contrast, male KO mice spent an equal amount of time exploring the familiar and novel object, indicating that they were unable to discriminate between the two objects (Bonferroni comparisons: **KO** *N vs. F*: *p* > 0.9999). Similarly, female KO mice showed an impaired recognition memory as they did not discriminate between the novel and familiar object on the testing day (Figure 1, two-way repeated measures ANOVA, *female*: F(1,28) = 4.246, *p* = 0.0495; Bonferroni comparisons: **KO** *N vs. F*: *p* > 0.9999). In contrast, female WT mice spent significantly more time exploring the novel object (Bonferroni comparisons: **WT** *N vs. F*: *p* = 0.0215). In addition, calculation of the discrimination index (DI) showed significantly higher values for both male and female WT animals compared to the respective KO groups on the testing day (Figure 1d,h; *t*-test, *male:* F(12,13) = 1.207, *p* = 0.0190; *female:* F(14,14) = 3.105, *p* = 0.0450). The distance traveled did not differ between WT and KO mice, regardless of sex (Figure 1c,g; *t*-test, *male:* F(12,13) = 2.592, *p* = 0.3543; *female:* F(14,14) = 1.409, *p* = 0.0504).

**Figure 2:**
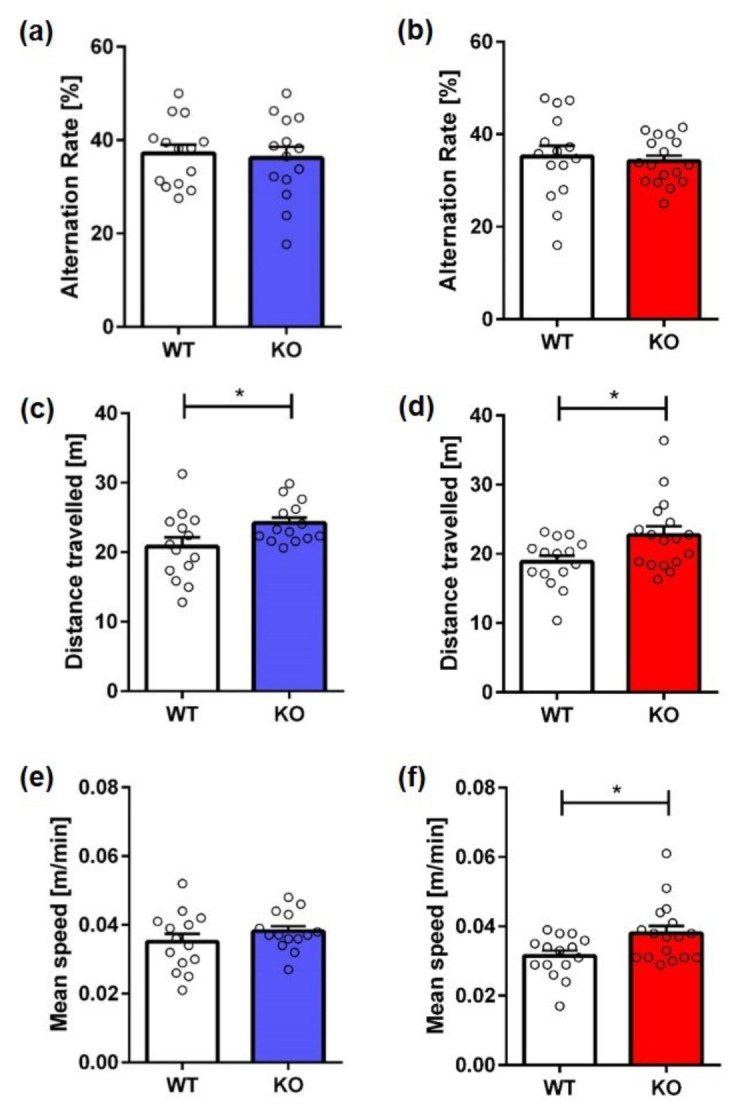
**Working memory and exploration behavior of Mover KO mice in the cross maze.** Spontaneous alternation did not differ between male (**a**) or female (**b**) KO mice and same-sex WT animals. Male (**c**) and female (**d**) KO mice traveled significantly further than WT animals. In addition, female KO mice (**f**) showed an increased speed compared to WT mice. Mean speed did not differ between male KO mice and WT animals (**e**). Unpaired *t*-test, *n* = 15–17. Data presented as mean ± S.E.M. * *p* < 0.05.

Working memory in KO mice was evaluated by analyzing their spontaneous alternation behavior in the cross maze. No significant difference was detected between KO and WT animals in spontaneous alternation independent of sex (Figure 2a,b; *t*-test, *male:* F(12,13) = 1.634, *p* = 0.7441; *female:* F(14,14) = 3.409, *p* = 0.7114). However, male and female KO mice traveled a significantly longer distance than same-sex control mice (Figure 2c,d; *t*-test, *male:* F(12,13) = 2.810, *p* = 0.0358; *female:* F(14,14) = 2.192, *p* = 0.0203). In addition, female KO mice moved significantly faster than WT mice (Figure 2e,f; *t*-test, *male:* F(12,13) = 2.306, *p* = 0.2705; *female:* F(14,14) = 2.017, *p* = 0.0182).

Spatial reference memory was tested in Mover KO mice and aged-matched WT mice in the MWM. Testing began with 3 days of cued training to rule out possible motor or sensory deficits that could affect the performance of mice. KO and WT mice showed progressively decreased escape latencies over time independent of their sex (data not shown; two-way repeated measures ANOVA, *days*: **female** F(2,56) = 38.17, *p* < 0.001; **male** F(2,50) = 22.48, *p* < 0.001). Swimming speed did not differ between WT and KO mice (data not shown; two-way repeated measures ANOVA, *genotype*: **female** F(1,28) = 0.1400, *p* = 0.9066; **male** F(1,24) = 0.7732, *p* = 0.7833). Overall, the cued training demonstrated that all mice had the ability to perform the test.

**Figure 3:**
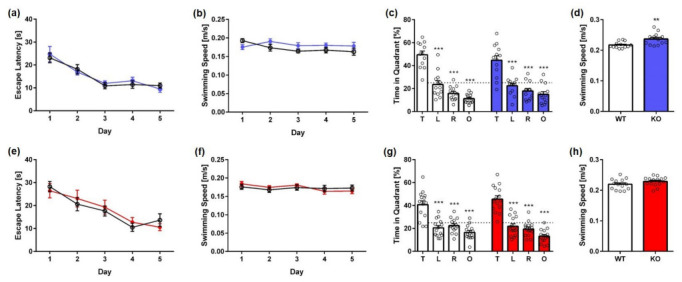
**Spatial learning and spatial reference memory of Mover KO mice in the Morris water maze.** Spatial learning of KO mice was not altered as mice improved significantly during acquisition training. Escape latencies did not differ significantly between male (**a**) or female KO mice (**e**) and WT animals. Swimming speed did not differ between male (**b**) or female (**f**) KO mice and WT animals in the acquisition training. In the probe trial (**c**,**d**), all animals displayed a clear preference for the target quadrant. Male KO swam significantly faster than WT animals (**d**). In contrast, swimming speed was not altered in female KO mice (**h**) in the probe trial. Chance level is indicated by a dashed line. Two-way (**a**,**b e**,**f**) and one-way (**c**,**g**) ANOVA followed by Bonferroni multiple comparisons and unpaired *t*-test (**d**,**h**); *n* = 13–15. Data presented as mean ± S.E.M. ** *p* < 0.01, *** *p* < 0.001.

During the subsequent acquisition training, the learning ability to locate a hidden platform using distal and proximal cues was tested. Across the 5 days of acquisition training, all animals, irrespective of sex and genotype, showed a significant decrease in the escape latencies (Figure 3a,e; two-way repeated measures ANOVA, *genotype*: **male** F(4,100) = 20.49, *p* < 0.001, **female** F(4,112) = 20.42, *p* < 0.001). Furthermore, the escape latency did not differ between KO and same-sex WT animals (two-way repeated measures ANOVA, *genotype*: **male** F(1,25) = 0.6434, *p* = 0.8018; **female** F(1,28) = 0.1299, *p* = 0.9101). Swimming speed did not differ between KO and WT animals during the acquisition training (Figure 3b,f; two-way repeated measures ANOVA, *genotype*: **male** F(1,25) = 2.666, *p* = 0.1151; **female** F(1,28) = 0.1299, *p* = 0.9101).

Twenty-four hours after the last day of acquisition training, a probe trial was performed to assess spatial reference memory. Both female KO and WT mice showed a significant preference for the target quadrant, as indicated by the relative time spent in the different quadrants of the pool (Figure 3g, one-way ANOVA followed by Bonferroni multiple comparisons, *quadrant preference:* **WT**: F(3,56) = 21.93, *p* < 0.001; Bonferroni for target vs. all other quadrants: *p* < 0.001, **KO**: F(3,56) = 44.50, *p* < 0.001; Bonferroni for target vs. all other quadrants *p* < 0.001). Similarly, male KO and WT mice showed a significant preference for the target quadrant in the probe trial (Figure 3c: one-way ANOVA followed by Bonferroni multiple comparisons, *quadrant preference:* **WT**: F(3,52) = 45.20, *p* < 0.001; Bonferroni for target vs. all other quadrants: *p* < 0.001, **KO**: F(3,48) = 23.92, *p* < 0.001). Swimming speed did not differ between female KO and WT mice in the probe trial (Figure 3h; unpaired *t*-test, *genotype:* female: F(14,14) = 1.357, *p* = 0.1234). In contrast, male KO mice showed an increased swimming speed compared to WT animals (Figure 3d; unpaired t-test, *genotype:* male: F(12,13) = 3.472, *p* = 0.0036).

In addition, the search strategies of mice during the acquisition training and probe trial were analyzed. The cognitive level of a particular search strategy can be quantified using a cognitive score that considers swim strategies based on their relevance to spatial learning. A higher cognitive score indicates primarily spatial learning, while non-spatial learning strategies such as “random search”, “scanning”, and “chaining” result in a low cognitive score.

**Figure 4:**
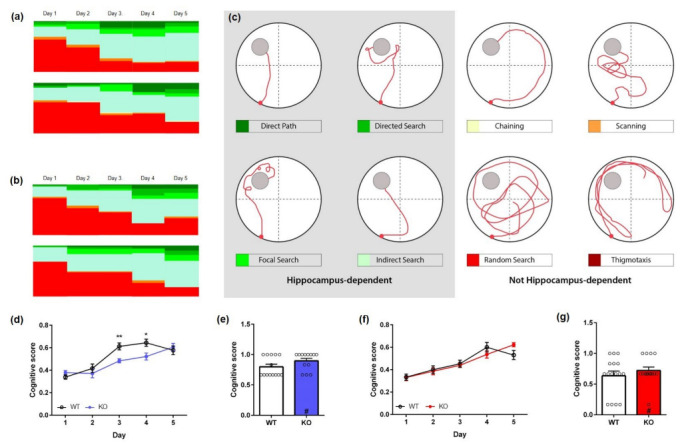
**Qualitative analysis of spatial learning of Mover KO mice in the Morris water maze.** Distribution of search strategies used by female (**a**) and male (**b**) KO Mover and same-sex WT mice. Animals showed a clear progression towards increasing spatial strategies over the 5 days of acquisition training. (**c**) Search strategies used by mice to locate the hidden platform in the MWM can be divided into hippocampus-dependent and non-hippocampus-dependent strategies. During the acquisition training, the cognitive scores of male (**d**) KO mice were significantly lower than those of WT animals. In contrast, cognitive scores did not differ between female KO and WT mice (**f**). During the probe trial, male (**e**) and female (**g**) KO mice showed a similar cognitive score compared to WT mice. Two-way repeated measures analysis of variance (ANOVA) followed by Bonferroni multiple comparisons (**d**,**f**) ANOVA and unpaired *t*-test (**e**,**g**); *n* = 13–15. Data presented as mean ± S.E.M. ANOVA: * *p* < 0.05, ** *p* < 0.01 (genotype difference); # *p* < 0.05 (sex difference).

During the first day of acquisition training, both male KO and WT animals predominantly used a “random search” strategy (Figure 4a; chi-square, *genotype:* **Day 1**: *p* = 0.5995). As training progressed, non-spatial search strategies decreased in both WT and KO mice. However, WT animals shifted more quickly to spatial strategies, as non-spatial strategies were almost absent by day 4 of acquisition training (17%). In contrast, KO mice continued to predominantly use a “random search” strategy (41%) until day 4 (chi-square, *genotype:* **Day 4**: *p* = 0.0091). During the last day of acquisition training, the search strategies did not differ significantly between WT and KO mice (chi-square, *genotype:* **Day 5**: *p* = 0.4005) with both genotypes using a mixture of spatial search strategies. In addition, male KO mice showed a lower cognitive score than same-aged WT mice in the acquisition training (Figure 4d; two-way repeated measures ANOVA, *genotype*: **male** F(1,25) = 5.252, *p* < 0.01; Bonferroni multiple comparisons: Day 3, Day 4: *p* < 0.05).

In contrast, search strategies of female KO mice did not differ significantly from WT over the 5 days of acquisition training (Figure 4b; chi-square, genotype: **Day 1**: *p* = 0.5351, **Day 2**: *p* = 0.1522, **Day 3**: *p* = 0.6135, **Day 4**: *p* = 0.0964, **Day 5**: *p* = 0.2499). During the first day of acquisition training, both female KO and WT animals predominantly used a “random search” strategy (WT: 67%; KO: 68%). As training progressed, non-spatial search strategies decreased in both WT and KO mice. Furthermore, the cognitive score of female KO mice did not differ significantly from same-aged WT animals in the acquisition training (Figure 4f; two-way repeated measures ANOVA, *genotype:* F(1,25) = 0.0006, *p* = 0.9802).

During the probe trial, female and male KO and WT animals mainly relied on different forms of spatial search strategies (chi-square, *genotype:* **female**: *p* = 0.0133; **male**: *p* = 0.1440) and the cognitive score did not differ significantly between the genotypes (Figure 4e,g, unpaired *t*-test, *genotype:* **male** F(12,13) = 1.275, *p* = 0.1056; **female** F(14,14) = 2.017, *p* = 0.3710).

### 2.2. Reduced Anxiety Behavior in MOVER Knockout Mice

**Figure 5:**
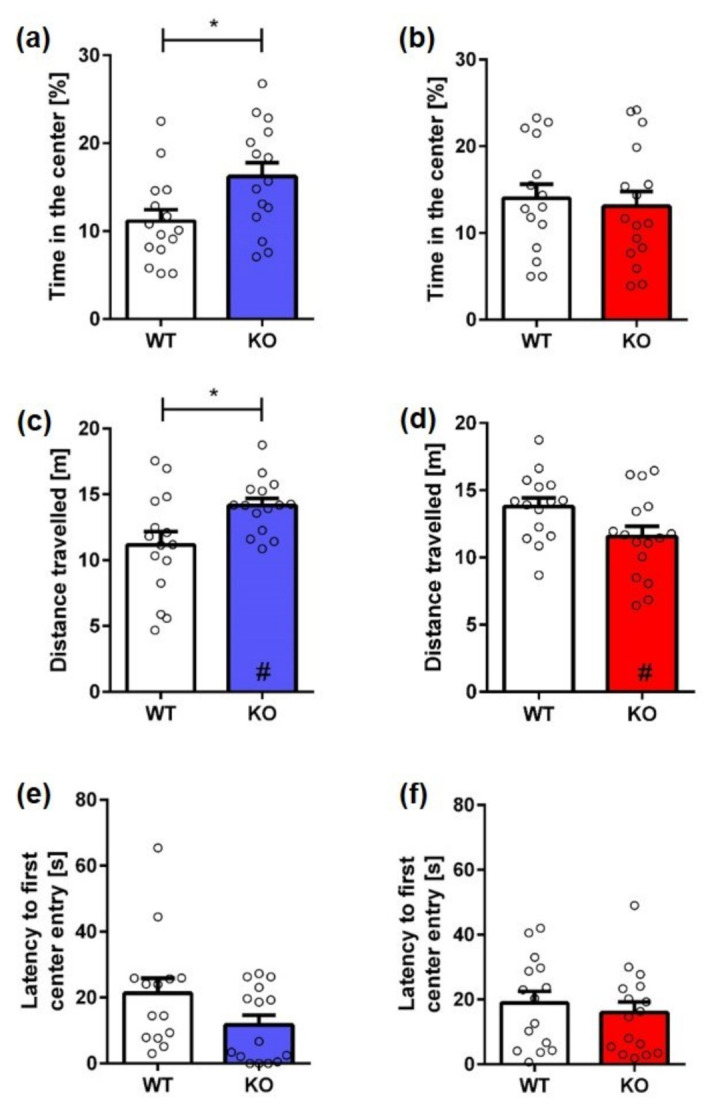
**Exploration and anxiety behavior of Mover KO mice in the open field.** Male KO mice spent more time in the center of the arena (**a**) and were more active than WT animals (**c**). In contrast, there was no significant difference in the time spent in the center of the box (**b**) or the distance traveled (**d**) between female KO and WT animals. The latency to first enter the center of the arena did not differ between KO and WT mice, independent of sex (**e**,**f**). Unpaired *t*-test, *n* = 15–17. Data presented as mean ± S.E.M. * *p* < 0.05 (genotype difference); # *p* < 0.05 (sex difference).

Exploratory and spontaneous locomotor activity of KO mice was compared to WT mice in the OF test. Male KO mice spent significantly more time in the center of the maze compared to their WT littermates, reflecting reduced anxiety (Figure 5a; unpaired *t*-test, *genotype:* **male**: F(12,13) = 1.514, *p* = 0.0187). In addition, male KO mice traveled further than WT mice (Figure 5c; unpaired *t*-test, *genotype:* **male**: F(12,13) = 2.967, *p* = 0.0271). Furthermore, male KO mice traveled significantly more than female KO mice (unpaired *t*-test, *sex:* **KO**: F(12,14) = 1.544, *p* = 0.0386). There was no significant difference in the time mice took to reach the center of the arena at the beginning of the experiment (Figure 5e; unpaired *t*-test, *genotype:* **male**: F(14,14) = 2.276, *p* = 0.0838).

In contrast, no significant difference was found between female KO and WT mice in terms of time spent in the center (Figure 5b; unpaired *t*-test, *genotype:* **female**: F(14,14) = 1.149, *p* = 0.6998). Furthermore, the latency to reach the center of the arena (Figure 5f; unpaired *t*-test, *genotype:* **female**: F(14,14) = 1.145, *p* = 0.5492) and the distance traveled did not differ between female KO and control mice (Figure 5d; unpaired *t*-test, *genotype:* **female**: F(14,14) = 1.956, *p* = 0.1141).

**Figure 6:**
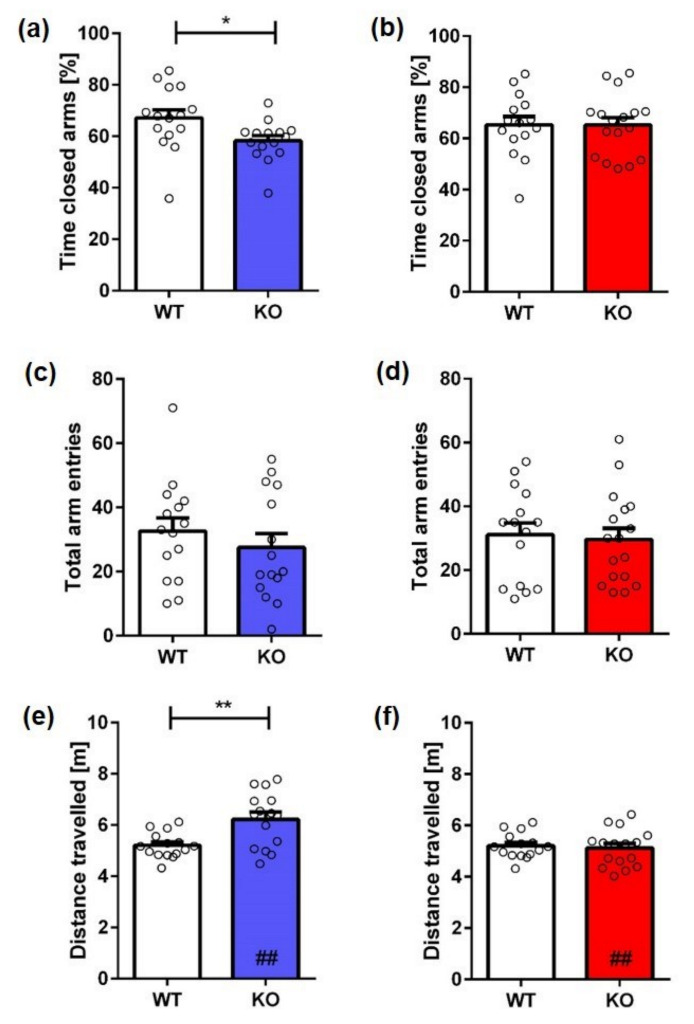
**Exploration and anxiety behavior of Mover KO mice in the elevated plus maze.** Male KO mice spent significantly less time in the closed arms of the maze (**a**) and traveled (**e**) significantly further than WT mice. In contrast, no significant differences in time spent in the closed arms (**b**) or distance traveled (**f**) were observed in female mice. The number of arm entries did not differ between male KO (**c**) or female KO (**d**) and same-sex WT animals. Unpaired *t*-test, *n* = 15–17. Data presented as mean ± S.E.M. * *p* < 0.05; ** *p* < 0.01 (genotype difference); ## *p* < 0.01 (sex difference).

The results in the EPM further corroborated the findings from the OF test. Male KO mice showed a decreased anxiety phenotype, which was indicated by spending less time in the closed arms (Figure 6a; unpaired *t*-test, *genotype:* **male**: F(12,13) = 2.517, *p* = 0.0276). Furthermore, male KO mice traveled significantly more than WT animals (Figure 6e; unpaired *t*-test, *genotype:* **male**: F(14,14) = 4.643, *p* = 0.0024), while the total number of arm entries was unchanged (Figure 6c; unpaired *t*-test, *genotype:* male: F(14,14) = 1.114, *p* = 0.3974). In addition, male KO mice traveled significantly more than female KO mice (unpaired *t*-test, *sex:* **KO**: F(15,14) = 2.210, *p* = 0.0020). In contrast, female KO mice spent a similar amount of time in the closed arms of the maze as their WT littermates (Figure 6b; unpaired *t*-test, *genotype:* **female**: F(14,14) = 1.014, *p* = 0.9932). Furthermore, the total number of arm entries (Figure 6d; unpaired *t*-test, *genotype:* **female**: F(14,14) = 1.015, *p* = 0.7575) and distance traveled (Figure 6f; unpaired *t*-test, *genotype:* **female**: F(14,14) = 2.101, *p* = 0.7035) were unaltered in female KO mice.

## 3. Discussion

The presynaptic protein Mover is absent in *Drosophila* and *C. elegans*, indicating that Mover is not essential for the basic and evolutionarily conserved core neurotransmitter release machinery. Instead, it may modulate neurotransmitter release at certain synapses, contributing to synaptic heterogeneity [8,9]. For example, deletion of Mover does not affect synaptic transmission at CA3 to CA1 synapses, but strongly increases short-term facilitation at mossy fiber (MF) to CA3 synapses, including frequency facilitation, a hallmark of mossy fiber terminal function [9]. To investigate the role of Mover on behavior, mice lacking Mover were subjected to a series of memory and anxiety tests.

Our key findings are that, first, Mover-deficient mice of both sexes lack recognition memory in the NOR task. Thus, the presence of Mover in mice is indeed required for normal interactions with the environment in mice. In addition, Mover is required for normal anxiety responses in male mice. Second, working memory, spatial learning, and spatial memory were not affected. Thus, Mover is specifically required for memory related to novel object recognition, but not for tasks involving spatial memory. Third, while novel object recognition was impaired in KO mice of both sexes, only male KO mice showed reduced anxiety and increased locomotion, and only male KO mice showed a change in the search strategy in the MWM compared to WT mice. Thus, the absence of Mover does indeed affect mouse behavior, is relevant for recognition memory and anxiety, and causes both sex-dependent and sex-independent phenotypes.

The hippocampus is crucially involved in learning and memory via its trisynaptic circuit. Short-term synaptic plasticity in the hippocampal CA3 region and MF synaptic transmission in this region are associated with memory and cognition [10,11,12]. While the prefrontal cortex (PFC) mainly encodes task-relevant information in working memory [13], the hippocampus also plays a crucial role in working memory [14]. In particular, the similar time regime of MF frequency facilitation and working memory, both of which occur on a time scale of seconds, has led to the suggestion that such facilitation could be the biological substrate of working memory [11,15]. Hence, the aberrant short-term synaptic plasticity in CA3 in Mover KO mice, evident as increased frequency facilitation at the MF terminals [9], led to the question of whether working memory is affected in Mover KO mice. We found here that working memory, as tested in the cross maze, is not affected in Mover KO mice. Thus, the increased frequency facilitation at hippocampal MT terminals does not affect working memory in these mice. On a more general level, this argues against a direct correlation between working memory and MF short-term plasticity.

Alterations in synaptic plasticity in the hippocampus often lead to impairments in spatial memory [16]. Mover KO mice showed a normal spatial memory performance in the MWM with respect to escape latency. Similarly, mice conditionally lacking Bassoon at the glutamatergic synapses of the hippocampus and neocortex showed no spatial learning or re-learning deficits [1]. In the hippocampus, selective knockout of Bassoon at the glutamatergic synapses of the hippocampus represents a similar situation compared to the knockout of Mover, because Mover is primarily at the glutamatergic synapses in the mouse hippocampus [7]. Thus, a lack of either Bassoon or Mover at the glutamatergic hippocampal synapses does not affect spatial learning. In contrast, mice lacking RIM1α—an active zone protein primarily expressed in the brain that is involved in several aspects of presynaptic function—revealed abnormalities in learning and memory in both MWM and fear conditioning [4,17,18]. Hence, while Mover and Bassoon, together with Munc13-1 and the scaffolding proteins CAST and Piccolo, may all be part of a complex including RIM1 [3,19,20], ablation of Mover or Bassoon affects learning and memory differently than ablation of RIM1α. Interestingly, a detailed analysis of the swimming strategies demonstrated allocentric-specific memory deficits in male KO mice. KO mice held on to non-spatial strategies longer than WT mice during the acquisition training displaying slight spatial navigation deficits. This deficit did not result in increased escape latencies because the non-spatial search strategies employed by male KO mice were suffice to find the platform, but it nonetheless represents a behavioral phenotype.

Here, we were able to demonstrate for the first time that Mover seems to play a crucial role in recognition memory in the NOR as both male and female Mover KO mice were unable to distinguish between a new and familiar object. In contrast to other memory tests that can be clearly linked to a specific brain region, the object recognition test seems to rely on multiple brain regions and neurotransmitter systems, including the hippocampus and perirhinal regions, making it particularly difficult to interpret in terms of underlying neurobiology [21,22,23,24,25,26]. In contrast to Mover KO mice, Bassoon KO mice displayed an increased novelty preference in a spatial discrimination/pattern separation task similar to the NOR test [1]. These changes were associated with an increase in baseline synaptic transmission at the synapses of the medial perforant path to the dentate gyrus. Bassoon is a large scaffolding protein. If the small protein Mover, by binding to Bassoon, was recruited to the active zones to mediate some of the functions of Bassoon, knockout of Mover should cause at least part of the phenotypes associated with the knockout of Bassoon. The increased novelty preference observed in Bassoon KO mice compared to the decreased novel object recognition memory in Mover KO mice makes it unlikely that Mover acts downstream of Bassoon to mediate its functions. Rather, Mover may inhibit these functions of Bassoon or act independently, without physically interacting with Bassoon. Interestingly, Nitta et al. (2021) demonstrated that downregulation of Piccolo, a presynaptic scaffolding protein structurally related to Bassoon, in the medial prefrontal cortex reduced recognition memory in the NOR [27].

Mover KO mice displayed increased exploratory behavior and increased locomotor activity, which is often a sign of decreased anxiety [4,28]. Indeed, the open field test and the elevated plus maze confirmed an anxiolytic effect upon the absence of Mover in male mice. This is well in line with several studies linking synaptic plasticity in the hippocampus to anxiety [16,29]. However, it has to be noted that, while anxiety can be linked to synaptic plasticity in the hippocampus, other brain regions also play an important role in anxiety behavior. Furthermore, the sex-specific effects regarding anxiety may be linked to regions in the hypothalamus that often have sex-specific functions [30] and need to be further assessed in future studies. Moreover, in contrast to the distribution in the hippocampus, Mover levels are uniformly high in the amygdala, a part of the brain that controls emotions [7]. It is involved in fear conditioning, fear and anxiety evaluation, and emotional and sexual behavior [31,32]. Independent of the site of action, Mover is important to allow for proper anxiety responses. The link between the expression of Mover and anxiety is not the first to connect this protein to a psychiatric disorder: Mover has been shown to be strongly upregulated in the brains of schizophrenic patients [33]. Interestingly, other synaptic proteins, such as SNARE proteins, have also been implicated in schizophrenia and the respective mouse models show an anxiety phenotype [34]. In addition, the effects of downregulating Piccolo in the medial prefrontal cortex, as discussed above in the context of novel object recognition, were proposed to represent schizophrenia-related behavior [27].

Furthermore, it has been proposed that runaway excitation, possibly due to glutamate spillover, is a prominent feature of many psychiatric disorders such as schizophrenia [35]. This highlights the appeal of future studies addressing the role of Mover not only in synaptic transmission but also in pathophysiology, as Mover might have evolved to buffer synaptic strength and avoid runaway neurotransmitter release.

Taken together, our data demonstrate for the first time that Mover appears to control specific forms of memory formation and behavior. Mover KO mice show impaired recognition memory and decreased anxiety behavior, suggesting that it has a modulatory role in synaptic transmission.

These considerations should be taken with caution because a complete picture of how presynaptic short-term plasticity, long-term plasticity, or presynaptic signaling are involved in learning and memory is not yet established. Until recently, relatively few studies have examined the role of presynaptic proteins in learning and memory in complex systems and this is the first study to investigate the influence of the absence of the presynaptic protein Mover in well-established behavioral tests. It will be important to further understand how presynaptic proteins are modified during synaptic plasticity and during learning tasks in vivo. Therefore, future functional studies involving knockout and knockin models of Mover, employing electrophysiological and biochemical methods, are required to analyze the presynaptic pathways modulated by Mover.

## 4. Materials and Methods

### 4.1. Mover Knockout Mice

The global Mover knockout (KO) line was generated as previously described [9,36]. In brief, conditional KO mice carrying loxP sites upstream of exon 1 and downstream of exon 3 of Mover were crossed with mice expressing Cre recombinase under the E2A promoter to generate a global Mover KO [9]. The KO was verified by sequencing and Western blotting. After removing Cre by breeding, mice were back-crossed to C57BL/6J for more than eight generations. In this study, male and female 8-week-old KO and wildtype (WT) control mice were used (WT: male *n* = 14, female *n* = 15; KO: male *n* = 13, female *n* = 15). For genotyping, WT and KO animals were identified by the presence of a specific 697-base pair (bp) and an 867-bp band, respectively.

Mice were housed in individually ventilated cages in a controlled environment on a 12/12 h light/dark cycle in groups randomly divided up to five. Water and food were available ad libitum. All animals were handled according to the German guidelines and EU legislation for animal care and the experiments were approved by the local authorities (Niedersächsisches Landesamt für Verbraucherschutz [17/2631]). All experiments followed the recommendations in the ARRIVE guidelines, and experimenters were blinded to the genetic status of the mice.

### 4.2. Behavior Testing

To detect possible behavioral and cognitive alterations in Mover knockout mice, animals were tested in a battery of memory, motor, and anxiety tests. All mice were analyzed at the age of 2 months and testing lasted 18 days. All behavior experiments were performed during the dark phase between 7 a.m. and 7 p.m.

### 4.3. Elevated Plus Maze

The elevated plus maze (EPM) was used to assess exploratory and anxiety-related behavior [37]. The apparatus was made of four arms (5 cm width × 15 cm length) extending at 90° angles from a central area (5 cm width × 5 cm length) raised 75 cm above a padded surface. The maze consisted of two oppositely positioned enclosed arms that were surrounded by a 15 cm high translucent plastic wall on three sides and two open arms [38]. Mice were placed in the center facing one of the open arms and were allowed to freely explore the maze for 5 min. Time percentage spent in each arm, distance traveled and total arm entries were recorded using the ANY-Maze tracking software (Stoelting Co, Wood Dale, IL, USA). Anxiety-like behavior was calculated based on the time spent in the open arms, with longer times spent in the open arms corresponding to lower levels of anxiety [39]. The EPM was cleaned after each mouse using 70% ethanol to eliminate odor cues.

### 4.4. Open Field

Explorative behavior and spontaneous motor activity were analyzed using the open field (OF) test [40]. Mice were tested for 5 min in a 50 × 50 cm arena with 38 cm high walls. ANY-Maze video tracking software (Stoelting Co, Wood Dale, IL, USA) was used to record time spent in the center area as well as the distance traveled. Between mice the maze was cleaned with 70 % ethanol to diminish odor cues.

### 4.5. Novel Object Recognition Task

The novel object recognition (NOR) is a commonly used behavioral task to evaluate recognition memory and novelty preference [41]. Twenty-four hours after the OF, NOR was performed in the same box, now containing two identical objects. Mice were allowed to freely explore the objects for 5 min. Twenty-four hours later, one of the two objects was replaced by a novel object similar in height but different in shape and appearance (testing phase). The ANY-Maze video tracking software (Stoelting Co., Wood Dale, IL, USA) was used to record the distance traveled and the exploration time of each object during a single 5 min trial on both days.

The percentage of exploration time for the novel object was calculated as follows:

$$\mathrm{Novel}\;\mathrm{Object}\;\left[\%\right]=\left(\frac{\mathrm{Novel}\;\mathrm{Object}}{\mathrm{Novel}\;\mathrm{Object}+\mathrm{Familiar}\;\mathrm{Object}}\times 100\right)$$

In addition, discrimination indices (DI) were calculated as follows:

$$\mathrm{DI}=\left(\frac{\mathrm{Time}\;\mathrm{at}\;\mathrm{Novel}\;\mathrm{Object}-\mathrm{Time}\;\mathrm{at}\;\mathrm{Familar}\;\mathrm{Object}}{\mathrm{Total}\;\mathrm{Exploration}\;\mathrm{Time}}\right)$$

The objects and the box were cleaned with 70 % ethanol between each mouse to remove any lingering scents.

### 4.6. Cross Maze

Working memory was assessed by analyzing the spontaneous alternation behavior of mice in the cross maze [42,43]. The cross maze consists of four arms (30 cm length × 8 cm width × 15 cm height) arranged in a 90° position extending from a central region (8 cm length × 8 cm width × 15 cm height). During a single 10 min test session, each mouse was randomly placed in one arm, and allowed to freely explore the maze. Alternation was defined as successive entries into the four arms in overlapping quadruple sets (e.g., 1, 3, 2, 4 or 2, 3, 4, 1 but not 1, 2, 3, 1) [42]. The alternation percentage was calculated as the percentage of actual alternations to the possible number of arm entries. The ANY-Maze video tracking software (Stoelting Co, Wood Dale, IL, USA) was used to record the alternation rate, distance traveled, and speed. To diminish odor cues, the maze was cleaned with 70% ethanol solution between mice.

### 4.7. Morris Water Maze

Spatial reference memory of mice was evaluated using the Morris water maze (MWM) as previously described [44]. In brief, the test relies on spatial cues to locate a submerged hidden platform (10 cm diameter) in a circular pool (110 cm diameter) filled with non-transparent tap water. The pool was divided into four virtual quadrants that were defined based on their spatial relationship to the platform: left (L), right (R), opposite (O), and target (T) quadrant, which contained the goal platform [45].

During the 3 days of cued training, the platform was marked with a triangular flag and mice were given 60 sec to find the submerged platform. Each mouse received four training trials per day with an average inter-trial interval of 15 min. Both the location of the platform and the position at which mice were introduced into the pool changed between trials.

Twenty-four hours after the last day of cued training, mice performed 5 days of acquisition training. For this part of testing, the flag was removed from the platform. In addition to the distal cues existing in the room, proximal visual cues were attached to the outside of the pool. The platform location remained stationary for each mouse throughout training. Mice were introduced into the pool from one of four predefined entry points [44]. The order in which these entry points were used varied between training days. Trials were conducted as during the cued training phase.

Twenty-four hours after the last acquisition trial, a probe test was performed to assess spatial reference memory. The platform was removed from the pool, and mice were introduced into the water from a novel entry point. Mice were then allowed to swim freely for 1 min while their swimming path was recorded.

The ANY-Maze video tracking software (Stoelting Co., Wood Dale, IL, USA) was used to record escape latency, swimming speed, and quadrant preference.

Searching strategies during the acquisition training and probe trial were analyzed with Pathfinder (Jason Snyder Lab, Vancouver, Canada) [45,46]. Eight possible swim strategies were differentiated (Figure 4c): “direct path” (Ideal path error [IPE] ≤ 1250 mm; Heading error ≤ 40°), “directed search” (time in angular corridor ≤ 70% of trial; distance covered ≤ 4000 mm; IPE ≤ 15,000 mm), “focal search” (distance to swim path centroid ≤ 30% of radius; distance to goal ≤ 30% of radius; distance covered ≥ 1000 mm and ≤4000 mm), “indirect search” (IPE ≤ 3000 mm; average heading error ≤ 360°), “chaining” (time in annulus zone ≥ 90% of trial; quadrants visited ≥ 4; area of maze traversed ≤ 40% of maze), “scanning” (area of maze traversed ≥ 5% and ≤20% of maze; average distance to maze center ≤ 60% of radius), “random search” (area of maze traversed ≥ 10% of maze), and “thigmotaxis” (time in full thigmotaxis zone ≥ 65% of trial; time in smaller thigmotaxis zone ≥ 35% of trial; total distance covered ≥ 4000 mm). The different spatial parameters were adjusted to the experimental setup (goal position [x/y]: 275, 775; goal diameter: 200; maze diameter: 1100; maze center [x/y]: 550, 550; angular corridor width: 40; chaining annulus width: 200; thigmotaxis zone size: 50). Spatial strategies included “direct path”, “directed search”, “focal search”, and “indirect search”. “Chaining”, “scanning”, “random search”, and “thigmotaxis” were considered as non-spatial strategies (Figure 4c) [45].

Cognitive performance in the acquisition training was evaluated using a scoring system [45] in which higher cognitive strategies received higher scores: thigmotaxis = 0; random search = 1; scanning = 2, chaining = 3; indirect search = 4; focal search = 4; directed search = 5; direct path = 6. The average cognitive score was calculated for each mouse per day and normalized to six, the highest possible score.

### 4.8. Statistical Analysis

Differences between groups were tested with unpaired t-test, one-way analysis of variance (ANOVA) followed by Bonferroni multiple comparison or two-way analysis of variance (ANOVA) followed by Bonferroni multiple comparisons as indicated. For comparison of search strategies between groups, chi-square analysis was performed. Significance levels were defined as follows: *** *p* < 0.001, ** *p* < 0.01, * *p* < 0.05. All data were analyzed using GraphPad Prism 9.1.2 (GraphPad Software, San Diego, CA, USA).

## Data Availability

The data included in this study are available upon request.
